# Synergistic Enzybiotic Effect of a Bacteriophage Endolysin and an Engineered Glucose Oxidase Against *Listeria*

**DOI:** 10.3390/biom15010024

**Published:** 2024-12-28

**Authors:** David Talens-Perales, José-Antonio Daròs, Julio Polaina, Julia Marín-Navarro

**Affiliations:** 1 Instituto de Agroquímica y Tecnología de Alimentos, Consejo Superior de Investigaciones Científicas (IATA-CSIC), 46980 Paterna, Valencia, Spain; d.talens@iata.csic.es (D.T.-P.); jpolaina@iata.csic.es (J.P.); 2 Instituto de Biología Molecular y Celular de Plantas, Consejo Superior de Investigaciones Científicas, Universitat Politècnica de València, 46022 Valencia, Valencia, Spain; jadaros@ibmcp.upv.es; 3 Departmento de Bioquímica y Biología Molecular, Universitat de València, 46100 Burjassot, Valencia, Spain

**Keywords:** amidase, cell wall, hydrogen peroxide, antimicrobial, antibacterial, food safety

## Abstract

*Listeria monocytogenes* represents one of the main risks for food safety worldwide. Two enzyme-based antimicrobials (enzybiotics) have been combined in a novel treatment against this pathogenic bacterium, resulting in a powerful synergistic effect. One of the enzymes is an endolysin from *Listeria* phage vB_LmoS_188 with amidase activity (henceforth A10), and the other is an engineered version of glucose oxidase from *Aspergillus niger* (GOX). Both enzymes, assayed separately against *Listeria innocua*, showed antibacterial activity at the appropriate doses. The combination of the two enzybiotics resulted in a synergistic effect with a log reduction in viable cells (log N_0_/N) of 4, whereas, taken separately, the same dose of A10 and GOX caused only 1.2 and 0.2 log reductions, respectively. Flow cytometry and microscopy analyses revealed that A10 treatment alone induced the aggregation of dead cells. *L. monocytogenes* showed higher resistance to single treatment with GOX or A10 than *L. innocua*. However, the synergic combination of A10 and GOX resulted in a high lethality of *L. monocytogenes* with a log N_0_/N higher than 5 (below the detection limit in our analysis). Altogether, these results represent a novel efficient and eco-friendly antimicrobial treatment against the most lethal food-borne pathogen.

## 1. Introduction

Poisoning caused by the ingestion of food infected by *Listeria monocytogenes* is a serious cause of concern worldwide. This is best shown by the list of *Listeria* outbreaks that have occurred in recent years, reported by the USA Centers for Disease Control and Prevention (https://www.cdc.gov/listeria/outbreaks/, accessed on 15 April 2024). *L. monocytogenes* is a pathogen that can survive and grow under a wide range of rather extreme environmental conditions, including refrigeration temperatures and high salt concentrations [1,2]. This resilience enables the bacterium to persist in various food processing environments. *L. monocytogenes* can contaminate a wide range of food products, including raw and processed meats, unpasteurized dairy products, fresh products, and ready-to-eat foods. This bacterium can enter the food supply chain through various routes, such as soil, water, animals, and human carriers [3]. Contamination can occur during food production, processing, distribution, and handling [4]. Because of the ubiquity of *Listeria*, risk assessment models advise the implementation of effective cleaning and sanitation measures all along the food chain [5].

Cleaning and disinfection constitute an essential activity in all food industries and businesses at all levels, from manufacturing to retail premises. This is true in general terms and is particularly so in the case of resilient pathogens like *L. monocytogenes* [1]. Conventional disinfectants used in the food industry include chlorinated compounds, glutaraldehyde, alkylamines, alcohols and salts of quaternary ammonia. Although effective, these compounds pose serious environmental and health challenges as they can be highly toxic for humans [6,7,8]. Consequently, the search for alternative non-toxic, eco-friendly biocides has become a relevant biotechnological goal.

*Listeria innocua* has long been considered a useful non-pathogenic, surrogate model system for *L. monocytogenes*, tested for different studies in the food industry [9,10,11]. However, these species show different sensibilities to stresses such as heat, low pH or high salt concentrations, reflecting their genomic differences, despite their close genetic relationship [2,12]. These observations show the need to confirm potential deviations in the behavior of these species due to their different susceptibilities to any treatment.

Endolysins, bacteriophage-derived lytic enzymes that degrade the bacterial cell wall during the lytic cycle of phage infection, have become promising tools as new generation biocides [13]. These enzymes usually have a modular structure composed of a combination of enzymatically active domains (EADs) and cell wall binding domains (CBDs). EADs and CBDs are classified in different protein families with a wide diversity of catalytic activities or substrate specificities [14,15,16]. Besides their use as disinfectants, endolysins can also be used as food preservatives [17]. Because of their protein nature and very specific enzymatic activity against chemical bonds that sustain the bacterial cell wall, they do not confer negative effects, neither for the safety nor the organoleptic properties of the food product [17,18,19]. Endolysins are particularly suitable for use against Gram-positive bacteria, as is the case for the genus *Listeria*, whereas in Gram-negative bacteria, the existence of an outer membrane precludes the access of the enzyme to the cell wall [15]. In this respect, endolysins from phages that specifically attack *Listeria* species would be a preferred choice. This is the case for N-acetylmuramoyl-L-alanine amidases and glycosylhydrolases (GH25) [20,21,22,23]. As a drawback, the extent of endolysin action is somehow limited, and different endolysins show a saturation limit when their dose–response relationship is analyzed [20,22]. Interestingly, when endolysin PlyP40 activity was tested against different *Listeria* species, the susceptibility of *L. innocua* was in the range of *L. monocytogenes* strains, suggesting that some of these cell wall degrading enzymes have a broad-range specificity within this genus [21]. Since resilient *Listeria* cells that survive treatment may lead to new infection outbreaks, the exhaustive elimination of bacterial contamination is the objective of any antimicrobial strategy. On the other hand, the degradation of the bacterial cell wall caused by phage endolysins does not necessarily result in cell death, under specific conditions [24,25,26]. Indeed, *Listeria*, as well as other bacteria, is able to survive the action of endolysins by transitioning to a cell wall-deficient state that has been named L-form, which under a favorable environment, such as high-sugar or other osmo-protective media, can reproduce and even revert to its normal walled state [24,25,26]. Therefore, the application of endolysins can be useful to restrict *Listeria* proliferation in food environments but may not be sufficient for the effective eradication of the pathogen. To overcome this limitation, we aimed to assay the combined effect of an endolysin and an engineered version of glucose oxidase (GOX), a fungal enzyme with proven antimicrobial activity widely used in the food industry for different purposes [27]. The GOX biocidal effect is based on the production of hydrogen peroxide in the catalyzed reaction. Previous reports have shown that its use is less efficient against Gram-positive compared to Gram-negative bacteria, probably because the thicker peptidoglycan layer of the former increases the diffusion barrier for hydrogen peroxide [28,29]. Since *L. monocytogenes* is a catalase-positive and Gram-positive bacterium, high doses of GOX would be required to achieve efficient antibacterial action. We hypothesized that the hydrolysis of the cell wall with an endolysin would increase cell permeability to hydrogen peroxide, enhancing the effect of GOX. Indeed, our results show a clear synergy in the combined use of both enzybiotics against *Listeria*.

## 2. Materials and Methods

### 2.1. Enzyme Production and Quantification

The coding sequence of the A10 endolysin from *Listeria* phage vB_LmoS_188 (GenBank accession: AJE28029.1) was reverse-transcribed to cDNA and edited to improve expression in *Escherichia coli* using the Codon Optimization Tool provided by Integrated DNA Technologies (IDT) at https://eu.idtdna.com (accessed on 1 June 2022). To facilitate cloning into expression vector pQE-80L (Qiagen, Venlo, The Netherlands), the *Sac*I and *Pst*I restriction sites were incorporated at the 5′ and 3′ ends, respectively. Subsequently, the synthesized DNA fragment was digested with FastDigest *Sac*I and *Pst*I endonucleases (Thermo Scientific, Waltham, MA, USA) and inserted into the cloning vector using T4 DNA ligase (Thermo Scientific) to generate plasmid pA10. The endolysin coding region was fully sequenced to confirm the absence of mutations, and pA10 was used to transform competent Rosetta2 *E. coli* cells (MilliporeSigma, Burlington, MA, USA) for efficient protein production. Cell extracts were prepared from *E. coli* cultures grown at 37 °C until an optical density at 600 nm (OD_600_) of 0.6 is reached. Induction of gene expression was carried out by adding 1 mM IPTG (MilliporeSigma) followed by incubation at 16 °C 48 h. Cells were disrupted by sonication in Buffer A (20 mM sodium phosphate buffer, pH 7.4, 20 mM imidazole, 500 mM NaCl, 2% glycerol) and centrifuged at 12,000× *g* for 25 min. Purification of His-tagged A10 was carried out by nickel affinity chromatography, employing a 1 mL HisTrap FF column (Cytiva, Logan, UT, USA) mounted on an AKTA-Purifier (Cytiva). Elution was performed with buffer B (20 mM sodium phosphate buffer, pH 7.4, 500 mM imidazole, 500 mM NaCl, 2% glycerol). Eluted fractions containing A10 endolysin were dialyzed against buffer C (20 mM Tris-HCl, pH 7.0, 100 mM NaCl, 2% glycerol). Purity analysis of the recovered protein was evaluated by way of SDS-PAGE. Protein concentration was measured using a NanoDrop spectrophotometer (Thermo Fisher, Waltham, MA, USA).

In this study, we used an engineered version of glucose oxidase, with increased thermal resistance, produced in *Nicotiana benthamiana* [28]. This enzyme (GOX) was targeted to the plant apoplast, and extracted and dialyzed against buffer P (50 mM sodium phosphate pH 6), as previously described [28]. Since GOX was not pure in these fractions, the quantification of this enzyme was carried out by measuring its activity, expressed in mU/mL, where 1 U represents the amount of enzyme that releases 1 μmol of hydrogen peroxide in 1 min under the conditions of the assay. Activity assay was carried out as previously described [30]. Reagents in buffers A, B, C and P were purchased from MilliporeSigma.

### 2.2. Antibacterial Assays

The antibacterial activity of endolysin A10 and GOX was assayed against *Listeria innocua* CECT 910T- and *Listeria monocytogenes* CECT 940-type strains, which were obtained from the Spanish Type Culture Collection. Bacterial cultures were grown in liquid or solid TSBYE media (Tryptic Soy Broth with 0.6% Yeast Extract, both purchased from Condalab, Madrid, Spain) at 37 °C to an OD_600_ of 0.6. For assays in liquid medium, 300 μL of cells was collected by centrifugation at 12,000× *g* for 7 min, washed with 1 mL of filter-sterilized buffer P (50 mM sodium phosphate buffer pH 6) and finally resuspended with the same buffer to a final OD_600_ of 0.15 or 0.3.

Initially, the cells were subjected to single treatments, either with amidase A10 or GOX at different concentrations at 37 °C. The cells (80 μL; OD_600_ 0.15, equivalent to 9 log CFUs/mL) were either incubated with 80 μL of amidase A10 for 2 h or with 80 μL of a GOX mix, containing glucose and GOX in buffer P, for 90 min. In all cases, glucose was adjusted to a 2% (*w*/*v*) final concentration in the GOX mix. Finally, a two-step treatment was carried out, in which 80 μL aliquots of cells (OD_600_ 0.15) were initially incubated with 20 μL of amidase A10 to a final concentration of 6 μM for 30 min at 37 °C, and subsequently, 60 μL of the GOX mix was added, followed by a 90 min incubation at 37 °C. Control experiments were conducted by replacing each of the enzymes with buffer P. A sample from each treatment was transferred to 300 μL of TSBYE in a 96-multiwell plate, resulting in a final 1/500 dilution, and incubated at 37 °C. Growth was monitored by measuring the OD_600_ every 30 min using a microplate reader (SPECTROstar Omega, BMG LABTECH, Ortenberg, Germany). A logistic model was fitted to the growth curve, as previously described [28], to quantify the relative initial number of viable cells after each treatment (N). By comparison with the relative initial number of cells in the control treatment without enzymes (N_0_), the log N_0_/N was calculated. For assays on solid medium, 40 μL cells in buffer P (OD_600_ 0.3) were incubated with 20 μL of amidase A10 at a final concentration of 30 μM, for 30 min at 37 °C, before the addition of 100 μL of GOX mix containing GOX at 8 U/mL (final concentration) and further incubated for 90 min at 37 °C. Serial dilutions (1/10) were carried out, where 5 μL of each was spotted on TSBYE-agar medium and grown at 37 °C for 16 h.

### 2.3. Flow Cytometry

Control or treated cell samples were suspended in 160 μL of 0.8% NaCl, to which 40 μL of a solution of Syto9 Acid Nucleic Stain (SG) and propidium iodide (PI) was added to a final concentration of 6.7 µM and 40 µM, respectively, following the instructions of the manufacturer (LIVE/DEAD BacLight bacterial viability kit, Thermo Scientific, Cat#L7012), and incubated for 15 min at room temperature before observation. Flow cytometry was conducted using a MACSQuant16 Analyzer (Miltenyi Biotec GmbH, Bergisch Gladbach, Germany) equipped with MACSQuantify Software 2.13 (Miltenyi Biotec GmbH). The B1 channel (488 nm–525/50) was employed to analyze the SG dye, while the B3 channel (488 nm–615/20 nm) was used for the PI dye. The results obtained were analyzed using Floreada.io software (https://floreada.io/, accessed on 9 July 2024). Gates were set manually based on the control sample. A threshold signal higher than 10 was fixed for SG-positive (SG+) cells, covering 95% of the control cells. The SG+/PI-gate, corresponding to live cells, was established as a region corresponding to a relatively low incorporation of PI compared to SG within the SG+ region, as previously described [31,32]. The complementary region was set as SG+/PI+, labeling dead cells.

### 2.4. Microscopy Analysis

For microscopy analysis, the cells were stained using the same procedure as in flow cytometry. Aliquots (5 μL) of the cells were placed onto a glass slide and covered with a 0.17 mm thick coverslip. Samples subjected to the two-step treatment with A10 and GOX or control samples incubated with buffer were additionally stained with 1 μL of calcofluor (1%) dye (MilliporeSigma). Immersion oil type-a (mxa20234, Nikon) was applied over the coverslip before observation. Optical microscopy images were captured using an Eclipse 90i fluorescence microscope (Nikon) equipped with a 5-megapixel cooled digital color camera Nikon Digital Sight DS-5Mc (Nikon Corporation, Tokyo, Japan). Phase contrast illumination was employed, utilizing appropriate Nikon accessories (Ph3 condenser) and the CFI Plan Fluor DIC H/N2 100X Oil (MRH01900) objective. Fluorescent images were obtained using B-2E/C, G2-A and UV2-A Nikon filter blocks for SG, PI and CF, respectively, using the same 100× objective. The images were processed by using FIJI image software (version 2.16.1) [33].

### 2.5. Statistical Analysis

Statistical analysis was performed in R (version 4.3.0) [34]. To study the dose–response of each of the enzymes when applied separately, one-way ANOVA and post hoc Tukey analysis were carried out. To analyze the potential synergistic effect of the two enzybiotics, the response to the single and combined treatments was adjusted to a multiple linear regression model according to the following: y = β_0_ + β_1_ [Amidase] + β_2_ [GOX] + β_12_ [Amidase] [GOX], where [Amidase] represents A10 concentration in μM and [GOX] represents GOX concentration in U/mL.

## 3. Results and Discussion

### 3.1. Analysis of the Protein Structure of A10 Endolysin

**Figure 1:**
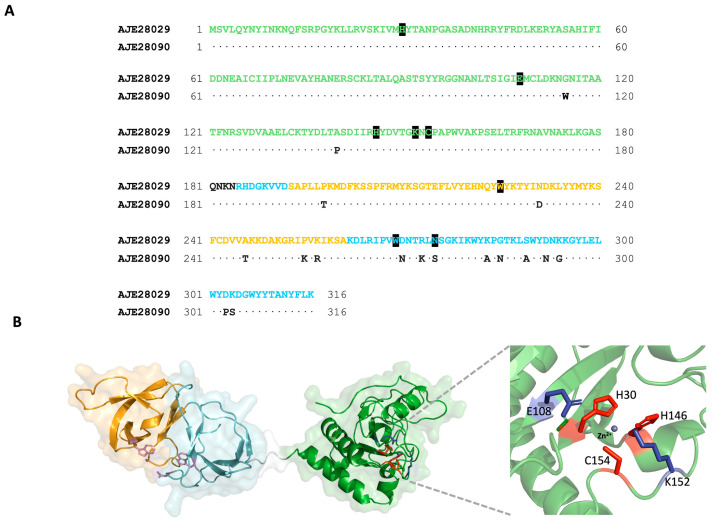
Sequence analysis of endolysin A10. (**A**) Sequence alignment of *Listeria* phage vB_LmoS_188 A10 (AJE28029) and *Listeria* phage vB_LmoS_293 (AJE28090). Residues building the EAD (green) and each of the SH3_b repeats (blue and orange) of the CBD domain are indicated. Residues forming the active site or involved in substrate binding in the CBD are highlighted in black. (**B**) Structural model of endolysin A10 created with Swissmodel [38]. Residues in the substrate binding cleft of the CBD are depicted in violet. A close-up of the active site is shown on the right with the general acid and base catalyst colored in purple and residues coordinating the Zn^2+^ ion in red.

Pennone et al. (2019) reported the cloning and analysis of the EAD domain of *Listeria* phage vB_LmoS_293 (293-amidase) [20]. This module showed muralytic activity and the capacity to prevent biofilm formation in *L. monocytogenes* on abiotic surfaces. BLAST analysis revealed the existence of another endolysin, *Listeria* phage vB_LmoS_188 (A10), with high identity (95%) to 293-amidase. Structural modeling and sequence alignment indicated that both enzymes are composed by an EAD belonging to the Ami-2 family of amidases (Interpro code IPR051206) at the N-terminal end (residues 1–180) and a CBD (Interpro code IPR041341) at the C-terminal end (residues 181–316), harboring most of the differences between the two proteins (Figure 1A).

In a survey of the Protein Data Bank, the catalytic domain of the amidase xlyA from *Bacillus subtilis* (3HMB) was the sequence that showed the highest identity (29%) to *Listeria* phage EAD [35]. A catalytic triad coordinating a Zn^2+^ ion, and the residues acting as the general acid and base, characteristic of the amidase domain [35], are clearly conserved in both sequences (Figure 1A,B). On the other hand, the best homologues to the CBD were the L-alanyl-D-glutamate peptidase from *Listeria* phage A500 (6HX0) and the amidase Ply protein from *Listeria* phage PSA (1XOV), with 78% and 80% sequence identity, respectively [36,37]. The CBD holds two SH3-b like domains, where the residues responsible for substrate binding are located at their interface [37]. Interestingly, these residues are also conserved in endolysin A10, but not in 293-amidase (Figure 1A,B). The mutation of any of these residues to alanine totally abolished the substrate binding capability of a homologous CBD [37]. For this study, the full-length version of endolysin A10 was used to preserve the function of the CBD domain.

### 3.2. Dose–Response Analysis of Endolysin A10 and GOX Against Listeria Innocua

**Figure 2:**
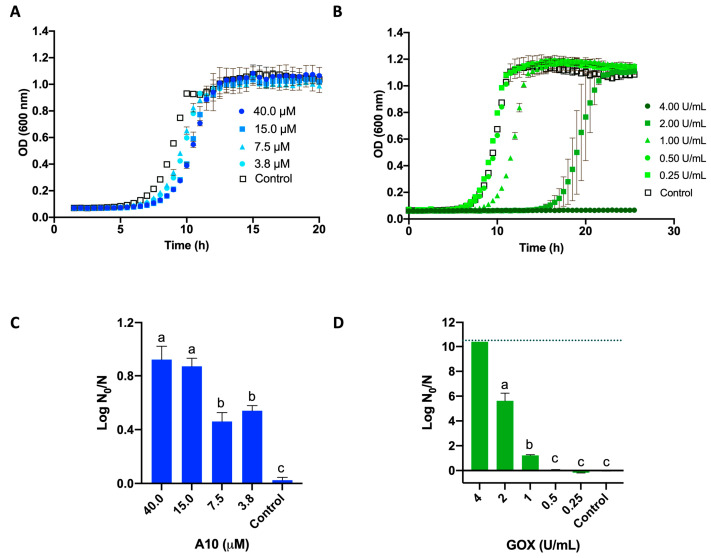
Antimicrobial activity of endolysin A10 (**A**,**C**) and GOX (**B**,**D**) against *L. innocua*. Log-phase cells were incubated with the enzybiotic at the expressed concentration for 2 h (A10) or 90 min (GOX), or buffer (control). Afterwards, liquid medium was inoculated and growth was monitored spectrophotometrically (**A**,**B**). The log N_0_/N ratio was quantified for each treatment by comparison with the corresponding control (**C**,**D**). Error bars indicate the standard deviation of duplicates. The dotted line in (**D**) indicates the detection limit (10.4 log N_0_/N). Different letters indicate significant differences between groups (ANOVA and Tukey post hoc test, *p* < 0.001; detailed information is shown in Tables S1 and S2).

The dose–response relationship of the antibacterial activity of A10 and GOX, applied separately, was analyzed as a first approach to establish a frame of experimental conditions in order to investigate a possible synergistic effect of both enzymes. Cell viability was monitored following the growth curve in a liquid medium after incubation with each one of the enzymes, compared to a control of untreated cells incubated only with buffer (Figure 2A,B). Longer lag phases would be expected as the initial number of viable cells was reduced. The relative number of viable cells after each treatment (N) or in the control (N_0_) was calculated using a logistic model to fit the growth curve data, as previously described [28]. The reduction in the number of viable cells compared to the control (log N_0_/N) shows that the GOX treatment follows a dose–response behavior up to the detection limit of the system (10.4 log reduction) (Figure 2D). In contrast, the A10 treatment reaches a maximum (ca. 1 log reduction) at 15 μM not surpassed at higher concentrations of enzyme (Figure 2C). This saturation of the antimicrobial effect has been previously reported in other endolysins [22]. Concentrations equal or below the saturation limit of A10 (15 µM) were selected for subsequent experiments. In the case of GOX, in order to avoid undesired side effects due to hydrogen peroxide production, low doses (below 0.25 U/mL), without significant antimicrobial effect in single treatment, were used.

### 3.3. Synergistic Biocidal Effect of Endolysin A10 and GOX Against Listeria Innocua

**Figure 3:**
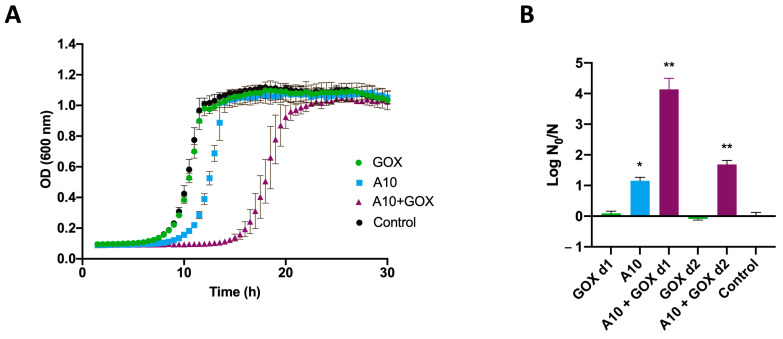
Synergistic antibacterial effect of endolysin A10 and GOX against *L. innocua*. (**A**) Cells were treated with A10 (6 μM final concentration) for 30 min, followed by the addition of GOX (0.25 U/mL final concentration) and further incubation for 90 min. To assay the individual effect of either A10 or GOX, incubations were performed in the same conditions, replacing each of the enzymes with buffer. The control sample was prepared likewise, replacing both enzymes with buffer. Subsequently, a liquid medium was inoculated with cells subjected to the different treatments, and growth was monitored spectrophotometrically. (**B**) The log N_0_/N ratio was quantified for each treatment by comparison with the control using the same amidase concentration (6 μM) and two different GOX concentrations (d1 = 0.25 U/mL and d2 = 0.06 U/mL). * indicates significant difference (*p* < 0.001) with the control; ** indicates significant interaction (*p* < 0.001) between the treatments (detailed information is shown in Table S3).

The combined effect of A10 and GOX was compared to the separate action of each enzyme (Figure 3). In these experiments, a two-step treatment was carried out with a previous A10 incubation for 30 min at 6 μM, followed by a GOX addition at doses of 0.25 U/mL or 0.06 U/mL for 90 min. As shown in Figure 3, the combined treatment with both enzymes caused a substantial decrease in bacterial viability, as revealed by a significant growth delay (Figure 3A). The log N_0_/N ratio of the combined treatment showed a synergistic effect compared to the single treatments (Figure 3B).

### 3.4. Flow Cytometry Analysis of Endolysin A10 and GOX Against Listeria Innocua

**Figure 4:**
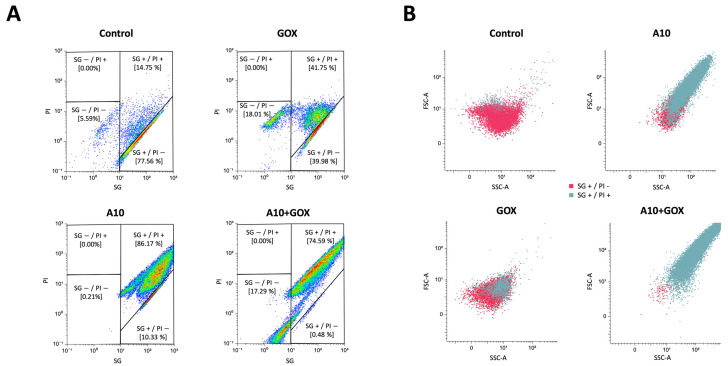
Flow cytometry analysis of *L. innocua* treated with endolysin A10 and GOX. Cells were treated with A10 (15 μM final concentration) for 30 min, followed by the addition of GOX (0.25 U/mL final concentration) and further incubation for 90 min. Single, double and control treatments are shown. (**A**) SG/PI analysis of the whole sample in each case. Selected gates to discriminate SG+/− and PI+/− populations are indicated. The percentage of cells in each gate is specified. Color coding represents lower (blue) and higher (red) event density. (**B**) SSC-A/FSC-A analysis of the SG+ population. SG+/PI+ and SG+/PI− cells are labeled with different colors.

The effect of the two enzymes, either used separately or in combination, on cell morphology and integrity was studied by flow cytometry. Cell viability was analyzed by the double labeling method with SG/PI (Figure 4A). The population of live cells (SG+/PI−) was reduced from ca. 80% in the control sample to 40% and 10% in the GOX and A10 single treatments, respectively. A synergistic effect was observed when both enzymes were combined, with a substantial reduction in live cells up to 0.5%. These results agree with those obtained by the analysis of the reduction in viable cells (log N_0_/N) after single or combined treatments (Figure 3B). Interestingly, when cells were incubated with GOX (either as a single or combined treatment), a significant increase in the SG population was observed (Figure 4A), which may be a consequence of a lower fluorophore stain due to DNA damage. A similar response has been reported with other chemical treatments generating radical species, such as ozonification or UV/chlorine. The induction of double-strand breaks or single-stranded regions has been proposed to explain this behavior [39,40,41] and may also occur as a result of hydrogen peroxide release by GOX. FSC-A/SSC-A analysis showed a remarkable effect on cell morphology as a result of the treatment with A10 amidase, with or without GOX, which was mainly associated with the population of dead cells (Figure 4B). The increase in FS, which is accompanied by an increase in SS, may be a result of cell aggregation as a consequence of peptidoglycan (PG) disassembly. This process was further studied by microscopy analysis.

### 3.5. Microscopy Analysis of Endolysin A10 and GOX Against Listeria Innocua

**Figure 5:**
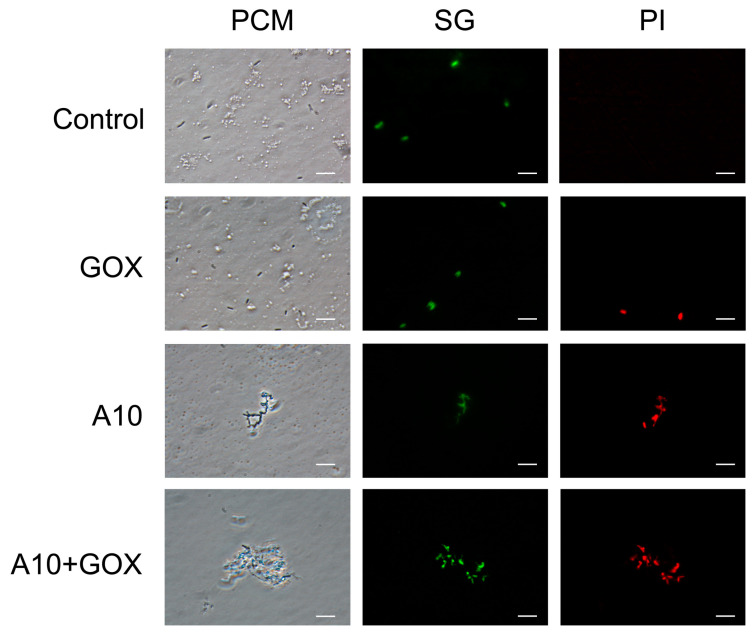
Microscopy analysis of *L. innocua* after different enzymatic treatments. Bacterial cells were stained with Syto9 (SG) and propidium iodide (PI) fluorophores and observed at 1000× under phase contrast microscopy (PCM) and fluorescence with two different filters (SG and PI). Scale bars correspond to 5 μm.

Microscopy analysis was conducted in order to confirm the morphology changes suggested by the flow cytometry analysis. Treatments with A10, either as a single enzyme or in combination with GOX, showed the presence of cell aggregates, which exhibited the double fluorophore labeling characteristic of dead cells (Figure 5). Single GOX treatment yielded the double labeling, indicating dead cells, but these were observed as separate, not aggregated cells. Observations under phase contrast microscopy suggest that the combined treatment of A10 and GOX results in a significantly increased disruption of the cellular structure compared to the A10 single treatment (Figure 5).

**Figure 6:**
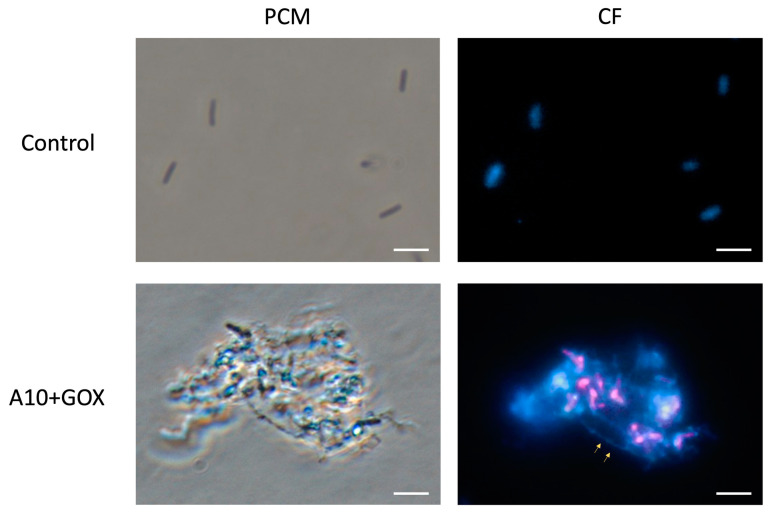
Microscopy analysis of *L. innocua* after combined treatment with endolysin A10 and GOX. Bacterial cells were stained with calcofluor and observed at 1000× under phase contrast microscopy (PCM) and fluorescence with UV filter (CF). Arrows indicate putative carbohydrate fibers resulting from PG disassembly. Scale bars correspond to 10 μm.

Staining with calcofluor, a compound which binds to cellulose and different β-glucans, including cellulose and chitin [42,43,44,45], was used to label the cell wall. Since the amidase cleaves the linkage between the polysaccharidic chain and the peptidic crosslink of the PG [35], it would be feasible to infer that enzymatic action releases carbohydrate fibers as those observed in Figure 6.

Bacterial aggregation similar to what we describe in this study has been previously reported for Gram-positive and Gram-negative bacteria treated with amidases [46,47]. Bacterial aggregation in Gram-negative was proposed to proceed by the insertion of the amidase in the outer membrane, coating the cell surface, prior to PG lysis. In Gram-positive bacteria, such as *Listeria*, such coating may occur directly on the PG layer of the cell wall, which is much thicker than in Gram-negative bacteria. Partially degraded cell wall polymers, together with some of the material released by initial cell lysis events, such as exogenous DNA [48], which acts as a scaffold matrix in bacterial biofilms, may cause cell aggregation.

Overall, microscopy analysis confirms the conclusions of the flow cytometry study, where a correlation was observed between cell aggregation and cell death. Aggregation does not occur by the clumping of live cells, as would be expected for a stress-induced defense mechanism. Rather, it seems a consequence of the endolysin treatment, subsequent to cell wall disintegration and cell death.

### 3.6. Synergistic Biocidal Effect of Endolysin A10 and GOX Against Listeria monocytogenes

**Figure 7:**
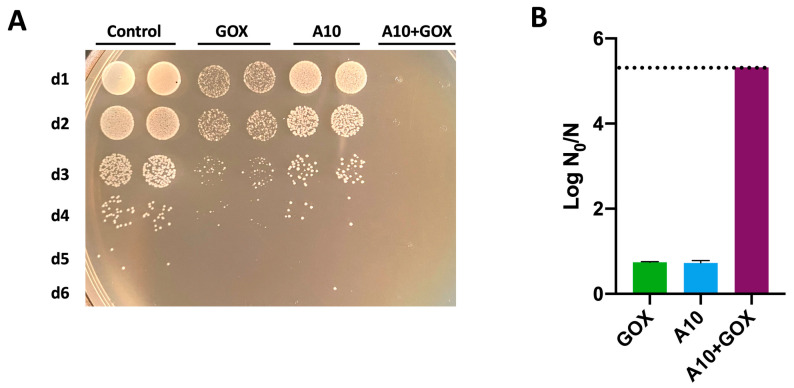
Synergistic antibacterial effect of endolysin A10 and GOX against *L. monocytogenes*. Cells were treated with A10 (30 μM final concentration) for 30 min, followed by the addition of GOX (8 U/mL final concentration) and further incubation for 90 min. To assay the individual effect of either A10 or GOX, incubations were performed in the same conditions, replacing each of the enzymes with the corresponding buffer. The control sample was prepared likewise, replacing the enzymes with buffer. Subsequently, serial dilutions (1/10) corresponding to d1 to d6 were carried out and spotted on LB-agar medium (**A**). The log N_0_/N for each treatment was calculated from the reduction in colonies compared to the control treatment (**B**). The dashed line in (**B**) represents the detection limit of the method.

The synergistic effect of the two enzymes was confirmed against *L. monocytogenes*. To this end, a semi-quantitative analysis was carried out using a spot test on solid media. Single treatments with A10 (30 μM) or GOX (8 U/mL) resulted in a 0.7 log reduction, whereas the combination of both enzymes caused at least a 5.3 log reduction (i.e., over the detection limit of the method), confirming the synergistic effect of the treatment observed with *L. innocua* (Figure 7). Thus, *L. monocytogenes* shows a higher resistance to single treatments of A10 or GOX than *L. innocua*. Indeed, a similar treatment with A10 caused a 0.9 log reduction in *L. innocua* (Figure 2C) and a lower dose of GOX (4 U/mL), resulted in a much higher (>10) log reduction (Figure 2D). The fact that amidase A10 can target both *Listeria* species, even though with different sensibilities, suggests a relatively broad substrate specificity within the *Listeria* genus. Although most of the cell wall-related genes from *L. monocytogenes* show a corresponding ortholog in *L. innocua*, a few genes are characteristic of each strain, which may reflect some differences in cell wall structure [12].

The results shown here point out the importance of combining the action of both enzybiotics to accomplish an effective elimination of *L. monocytogenes*, which is highly resistant to individual treatment with either enzyme. Despite a dose–response relationship found for GOX (Figure 2D), the combined strategy with two enzymes is clearly advantageous over a single treatment with higher doses of GOX, considering the potential non-specific side effects of the oxidative nature of the released hydrogen peroxide.

A similar methodology has been previously reported using bacteriophages combined with different disinfectant agents, including hydrogen peroxide, against *L. monocytogenes* [49]. However, extensive incubation times (at least 6 h) were required to achieve a 4-log reduction in colony forming units (CFUs) even when much lower cell densities (6 log CFUs/mL) were used in the inoculum, compared to that in this work (9 log CFUs/mL). The sensibility of bacteriophages to hydrogen peroxide may be a limitation to this method.

## 4. Conclusions

The antimicrobial (enzybiotic) effect of GOX due to the action of hydrogen peroxide generated as a catalytic product of its reaction is well documented [27,28]. However, the use of GOX for this purpose presents several limitations, such as the relative resistance of some catalase-positive *Listeria* strains to hydrogen peroxide or other oxidative treatments [50,51], side effects due to the oxidative nature of the peroxide and the requirement of glucose for the enzyme’s reaction. On the other hand, the effectiveness of endolysins as antibacterials has shown some limitations, such as a saturation limit in the dose–response, that constrain the efficacy of the treatment [20,22].

Another important issue related to antimicrobial treatments is the emergence of bacterial resistance. In the case of endolysin treatment, this is unlikely [15], particularly for endolysins with a relatively broad substrate specificity. This seems the case for A10, targeting both *L. innocua* and *L. monocytogenes*. On the other hand, a cell wall-deficient state derived from *Listeria* (L-form) has been described to survive after treatment with phages or endolysins [25], but only under osmo-protective media. Regarding resistances to GOX treatment, both *L. innocua* and *L. monocytogenes* are catalase-positive bacteria [12]. This activity decreases the concentration of hydrogen peroxide released by GOX. Although the mutation of *Listeria* to yield strains overproducing catalase is possible, the combination of two enzybiotics, with synergistic effects, based on different modes of action, makes the arousal of *Listeria* strains simultaneously resistant to both highly improbable.

In this study, we show that the combination of endolysin A10, an amidase produced by a bacteriophage specific for *Listeria*, and an engineered version of the GOX from *A. niger*, increases significantly the efficacy of the single treatments against *Listeria*. The synergistic effect of the enzybiotic combination shows that the constraints of each individual treatment can be surpassed by the addition of a complementary enzymatic activity.

## Data Availability

The original contributions presented in this study are included in this article. Further inquiries can be directed to the corresponding author.

## Supplementary Materials

The following supporting information can be downloaded at https://www.mdpi.com/article/10.3390/biom15010024/s1, Table S1: ANOVA and post hoc Tukey analysis of the dose–response study with A10; Table S2: ANOVA and post hoc Tukey analysis of the dose–response study with GOX; Table S3: Multiple linear regression analysis of the combined treatment with Amidase A10 and GOX.
