# Supplementary material for: Synergistic Enzybiotic Effect of a Bacteriophage Endolysin and an Engineered Glucose Oxidase Against Listeria
Source: Biomolecules. 2024 Dec 28;15(1):24. doi: 10.3390/biom15010024 (PMC11764271; doi:10.3390/biom15010024)
Supplement: Supplementary file 1 [file biomolecules-15-00024-s001.zip › biomolecules-3318426-supplementary.pdf]

Table S1. ANOVA and post-hoc Tukey analysis of the dose-response study with A10

<table>
<tr><td></td><td>Df</td><td>Sum of Sq</td><td>Mean Sq</td><td>F value</td><td>p (>F)</td></tr>
<tr><td>Group</td><td>4</td><td>1.0977</td><td>0.27442</td><td rowspan="2">66.79</td><td rowspan="2">0.000158</td></tr>
<tr><td>Residuals</td><td>5</td><td>0.0205</td><td>0.00411</td></tr>
<tr><td colspan="6">Post-hoc Tukey analysis</td></tr>
<tr><td colspan="6">“Control”; “3.8”;”7.5”;”15”;”40”
“a”;”b”;”b”;”c”;”c”</td></tr>
</table>

	Diff	Low	Upp	p adj
3.8-Control	0.5433042	0.28617762	0.8004308	0.0020272
7.5- Control	0.45798415	0.20085756	0.7151107	0.0044475
15- Control	0.87091413	0.61378755	1.1280407	0.0002111
40- Control	0.91764607	0.66051948	1.1747727	0.0001621
7.5-3.8	-0.08532005	-0.34244664	0.1718065	0.687833
15-3.8	0.32760993	0.07048334	0.5847365	0.0191616
40-3.8	0.37434187	0.11721528	0.6314685	0.0108806
15-7.5	0.41292998	0.1558034	0.6700566	0.0070773
40-7.5	0.45966192	0.20253533	0.7167885	0.0043744
40-15	0.04673194	-0.21039465	0.3038585	0.940509

Table S2. ANOVA and post-hoc Tukey analysis of the dose-response study with GOX

	Df	Sum of Sq	Mean Sq	F value	p (>F)
Group	4	48.16	12.041	157.1	$1.93 \cdot 10^{-5}$
Residuals	5	0.38	0.077		

Post-hoc Tukey analysis				
“Control”; “0.25”;”0.5”;”1”;”2”<br>“a”;”a”;”a”;”b”;”c”				
	Diff	Low	Upp	p adj
0.25-Control	-0.16723302	-1.27772068	0.9432546	0.9683461
0.5- Control	0.06384087	-1.04664679	1.1743285	0.9991417
1- Control	1.22939899	0.11891133	2.3398867	0.0338345
2- Control	5.62652624	4.51603858	6.7370139	0.000029
0.5-0.25	0.23107389	-0.87941377	1.3415616	0.9088332
1-0.25	1.39663201	0.28614435	2.5071197	0.0202215
2-0.25	5.79375926	4.6832716	6.9042469	0.000026
1-0.5	1.16555812	0.05507046	2.2760458	0.0416203
2-0.5	5.56268537	4.45219771	6.673173	0.0000304
2-1	4.39712725	3.28663959	5.5076149	0.0000946

Table S3. Multiple linear regression analysis of the combined treatment with Amidase A10 and GOX

	Estimate	Standard Error	t value	p value (> t)
Intercept	-0.04377	0.08292	-0.528	0.606
Amidase	0.18586	0.01954	9.510	$1.73 \cdot 10^{-7}$
GOX	0.46812	0.55861	0.838	0.416
Amidase:GOX	1.94907	0.13167	14.803	$6.06 \cdot 10^{-10}$
Residual standard error: 0.1786 on 14 degrees of freedom<br>Multiple R-squared: 0.9888; Adjusted R-squared: 0.9864<br>F-statistic: 412.2 on 3 and 14 DF, p value: $6.89 \cdot 10^{-14}$				
